# Advancing Survivors’ Knowledge (ASK) about skin cancer study: study protocol for a randomized controlled trial

**DOI:** 10.1186/s13063-015-0637-x

**Published:** 2015-03-24

**Authors:** Casey L Daniel, Gregory T Armstrong, Robyn R Keske, Jessica A Davine, Aaron J McDonald, Kim M Sprunck-Harrild, Catherine Coleman, Sebastien J Haneuse, Ann C Mertens, Karen M Emmons, Ashfaq A Marghoob, Elena B Elkin, Stephen W Dusza, Leslie L Robison, Alan C Geller

**Affiliations:** Department of Social and Behavioral Sciences, Harvard TH Chan School of Public Health, 677 Huntington Avenue, Kresge 718, 02115-6028 Boston, MA USA; Department of Epidemiology and Cancer Control, St. Jude Children’s Research Hospital, 262 Danny Thomas Place, MS 735, 38105-3678 Memphis, TN USA; Department of Medical Oncology, Dana-Farber Cancer Institute, 450 Brookline Ave, LW601, 02215-5450 Boston, MA USA; Department of Population Sciences, Dana-Farber Cancer Institute, 450 Brookline Avenue, 02215-5450 Boston, MA USA; Department of Biostatistics, Harvard TH Chan School of Public Health, 655 Huntington Avenue, Bldg 2, Rm 451, 02115-6009 Boston, MA USA; Department of Pediatrics, Emory University, Emory Children’s Center, 2015 Uppergate Drive, 4th floor, 30322-1014 Atlanta, GA USA; Kaiser Permanente, 1800 Harrison Street, 16th Floor, #161R03, 94612-3463 Oakland, CA USA; Memorial-Sloan Kettering Cancer Center, 1275 York Ave, 10065-6007 New York, NY USA; Department of Epidemiology and Biostatistics, Memorial Sloan-Kettering Cancer Center, 1275 York Avenue, Box 44, 10065-6007 New York, NY USA; Department of Medicine, Memorial Sloan-Kettering Cancer Center, 160 East 53rd St, 2nd Floor, 10022-5243 New York, NY USA

**Keywords:** Skin cancer, Early detection, Melanoma, Childhood cancer, Survivor, Dermoscopy, Skin self-examination, Health communication

## Abstract

**Background:**

Advances in treatment have increased childhood cancer 5-year survival rates to greater than 80%. However, children previously treated with radiation are at significantly increased risk of developing subsequent neoplasms, the most common of which are skin cancers. The National Cancer Institute and Children’s Oncology Group have issued recommendations for survivors treated with radiation to perform monthly skin self-examinations and receive a physician skin examination at least annually, as early detection has demonstrated markedly improved outcomes in the diagnosis and treatment of skin cancers. The goal of the present study is to increase rates of skin self-examinations and clinical skin examinations among adult survivors of childhood cancer treated with radiation.

**Methods/Design:**

This randomized controlled trial uses a 3-group comparative effectiveness design comparing: (1) *Patient Activation and Education* (PAE) including text messaging, print and web-based tutorials over 12 months; (2) *PAE plus physician activation* (PAE + MD) adding physician activation/educational materials about survivors’ increased skin cancer risk and conducting full-body skin exams; and (3) *PAE plus physician activation, plus teledermoscopy* (PAE + MD + TD) adding participant receipt of a dermatoscope intended to empower them to photograph suspect moles or lesions for review by the study dermatologist.

**Discussion:**

The current study addresses barriers to screening in this population by providing educational and motivational information for both survivors and physicians regarding the value of periodic skin examinations. It also utilizes innovative mobile health technology to encourage and motivate (that is activate) survivors to conduct skin self-examinations, request physician exams, and obtain treatment when worrisome lesions are found. Finally, as a comparative effectiveness trial, this study isolates the effects of adding specific components to the patient activation intervention to test the most effective intervention for enhancing skin examination vigilance among this high-risk group.

**Trial registration:**

Clinicaltrials.gov: NCT02046811; Registration date: 22 January 2014.

## Background

Advances in diagnosis and treatment for children with cancer have contributed to an overall 5-year survival rate that currently exceeds 80% [1]. There are over 420,000 estimated survivors of childhood cancer in the United States [2]. In a study of 1,713 childhood cancer survivors using clinical evaluations, Hudson *et al*. found that 95% of these individuals experienced at least 1 chronic health condition by age 45 [3,4]. The late effects of the treatment of childhood cancer includes impaired growth and development, decreased or loss of fertility, organ dysfunction, cognitive impairment [2,5,6], and second neoplasms which occur in an estimated 20.5% of these individuals [7-11]. Among survivors receiving radiation treatment, the relative risk of developing a subsequent neoplasm is 2.7 (CI = 2.2 to 3.3). These neoplasms most frequently occur within the radiation field [8].

Skin cancers, primarily basal cell carcinomas (BCCs), are the most common subsequent neoplasm faced by childhood cancer survivors [8,12,13]. Approximately 20 years after receiving radiation therapy, when most of these patients are in their 30s, this population faces the prospect of multiple and recurrent skin cancers at rates much greater than that of an age-matched general population [8,12-14]. Adult survivors of childhood cancer under 35 who were treated with radiation have nearly a 40-fold risk for non-melanoma skin cancer and more than 2.5 times the risk of melanoma compared with the general population [13,15,16]. Delay of identification and diagnosis of skin cancer can result in unnecessary morbidity [17]. Most recent evidence indicates that earlier diagnosis of a BCC can lead to smaller tumors, potentially less extensive treatment, better outcomes, and lower treatment costs [18].

Because of the extraordinarily high skin cancer rates in this special population, in April 2012 (updated in April 2014), the National Cancer Institute released a PDQ® (evidence-based data summary) strongly encouraging the use of the annual dermatological exam to screen for early-onset skin cancer in childhood cancer survivors [19]. The Children’s Oncology Group also specifies in its long-term survivorship guidelines that survivors of childhood cancer who received radiation should check their skin monthly for changes and have a thorough skin examination by a health care provider at least once a year [20].

### Importance of early detection

Skin cancer and its precursors ‘writes their message in the skin for all of us to see’ and can be easily seen by the patient, their providers, and significant others [21-25]. Therefore, teaching skin self-examination and encouraging patients to alert their physicians to skin changes provides a key opportunity for education and early detection. In the general population, thorough skin self-examination (TSSE), although only practiced by 15% of subjects, reduced mortality due to melanoma by an estimated 60% in one major case-control study [26]. Physician diagnosis was associated with a markedly higher rate of thinner melanoma in Australia [27], and a nearly 50% reduction in melanoma mortality in a region in Germany compared with unscreened control populations from adjacent regions in Germany and all of Denmark [28].

The American Academy of Dermatology has recommended the practice of skin self-examination to detect new and or changing lesions [29]. Individuals are encouraged to perform skin self-examinations regularly (for example, monthly) using the ABCDE (Asymmetry, Border, Color, Diameter, Evolution) algorithm [30]. Individuals should also request periodic full-body skin examinations as a national study of physicians indicate that they are more inclined to screen when requested to do so by their patients [31]. Such practices are particularly meaningful for those at highest risk for skin cancer, such as individuals treated with radiation during childhood. However, previous studies have demonstrated that only 29% of survivors report that they have ever received a physician skin examination for cancer [32].

## Methods/Design

### Study objectives and specific aims

The objective of this randomized study, entitled Advancing Survivors’ Knowledge (ASK) About Skin Cancer, is to determine the impact of a 12-month Patient Activation and Education intervention focused on early detection of skin cancer and timely medical follow-up among childhood cancer survivors treated with radiation. Key components of the ASK intervention utilize innovative technologies to activate patients to conduct skin self-examinations, request physician exams, and obtain treatment when worrisome lesions are found.

The specific aims of this study are: (1) to determine the impact of a Patient Activation and Education (PAE) intervention with and without physician activation (PAE + MD) and teledermoscopy (PAE + MD + TD) on skin cancer early detection practices measured at 12 and 18 months; (2) to determine the impact of the intervention on time to diagnosis; and (3) to estimate the cost and cost-effectiveness of the intervention. The intervention will teach skin cancer early detection skills to survivors, connect them with health care resources, and prompt medical staff who see this population to perform clinical skin exams.

### Study participants

The study will be conducted using the cohort of the Childhood Cancer Survivor Study (CCSS), funded by NCI grant U24 CA55727 (Principal Investigator, GT Armstrong). This cohort includes participants from 50 states diagnosed with a childhood cancer between 1970 and 1986 before age 21 years. Participants have been recruited by 27 clinical sites in the United States (US) and Canada. As of 2007, there were 12,323 known living participants, 46% of the sample were female, median age was 36 years, 73% were under age 40 years, 88% were White, non-Hispanic, and 25% had at least a college education [33-35].

Eligibility criteria for the ASK study include: (1) current age of 18 years or older; (2) previously treated with radiation for childhood cancer; (3) having seen their primary care physician or oncologist in the previous 2 years or planning to do so in the next year; (4) having no personal history of a skin cancer diagnosis, (5) possession of a cellular phone that can receive text messages, and (6) access to a DermLite (3Gen, San Juan Capistrano, CA, USA) compatible smartphone or tablet. Informed consent will be obtained from each enrolled participant in the ASK study.

### Conceptual model

The ASK study is guided by the 13-item Patient Activation Measure (PAM) [36], which posits that activated patients are better prepared to participate in self-management activities. Patient activation is increasingly seen as central to achieving improvements in the quality of care, better health outcomes, and less costly health care service utilization. Activation involves 4 stages: (1) believing that taking an active role as a patient is important, (2) having the confidence and knowledge necessary to take action, (3) actually taking action to maintain and improve one’s health, and (4) staying the course even under stress [36].

Individuals with higher levels of patient activation are more likely to engage in preventive behaviors such as attending routine check-ups and undergoing screening [37]. Utilizing key aspects of the PAM framework, the ASK study intervention was developed to emphasize the importance of understanding one’s role in managing his/her health and having the knowledge, skills, and confidence to do so.

Since 2004, numerous cross-sectional studies and randomized controlled trials have found patient activation to be related to healthy behaviors, appropriate use of the health care system, consumer behaviors (for example, researching physician qualifications, preparing a list of questions for a doctor visit), and chronic care self-management [38-41]. In an intervention trial of chronic disease patients, Hibbard and colleagues found positive change in activation resulted in positive change in various self-management behaviors [42]. Similarly, in a web-based randomized controlled trial for adults with asthma, hypertension, or diabetes, Solomon and colleagues determined that an online intervention had both positive and significant effects on the patient activation levels of participants in the intervention group (specifically attitudes toward knowledge, skills, and confidence in self-managing health), noting their increased likelihood to adhere to recommended health care practices [43].

Evidence suggests that primary care providers can play an important role in increasing patient activation [38,44]. For example, one study found that patients who report that their provider helped them learn to monitor their condition, set goals, and/or set up an exercise program, were more activated than patients who did not have this experience [38]. In addition to patient activation, the ASK study includes a primary care activation component described below.

### Design overview

**Figure 1:**
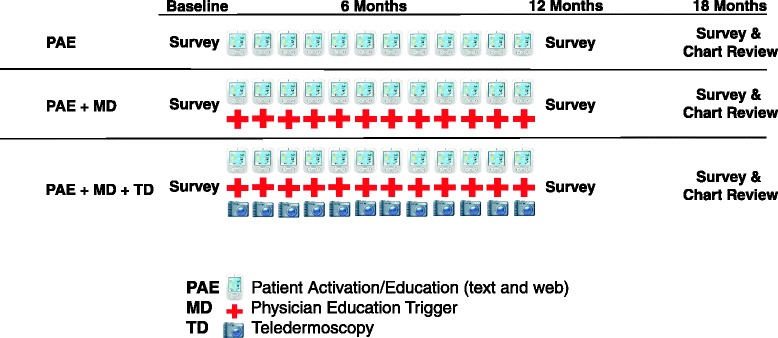
**Study design overview for the three intervention arms.**

This randomized controlled trial uses a three-group comparative effectiveness design comparing (Figure 1):

- *Patient Activation and Education* (PAE), including text messaging, web-based educational content, and print resources;
- *PAE plus physician activation* (PAE + MD): adding physician activation/educational materials (web and print) targeted to identified primary care providers;
- *PAE plus physician activation, plus teledermoscopy* (PAE + MD + TD): adding participant receipt of a dermatoscope and instructions for use. Teledermoscopy is a potentially valuable resource that may reduce costs and increase efficiency for both patients and physicians by providing expert skin assessments remotely [45].

### Primary study outcomes

Primary study outcomes will be measured at baseline, 12- and 18-months and include the following: (1) at least 1 thorough skin self-examination completed by the patient in the 2 months prior to both the 12- and 18-month survey; (2) at least 1 physician skin examination prior to 18-month survey; and, (3) reduction of the time interval between the first finding of a suspect lesion and a diagnostic visit.

## Procedures

### Eligibility screening

Participants of the CCSS cohort who received radiation for their childhood cancer and were not known to have a personal history of skin cancer are eligible to participate. Recruitment for the current intervention coincided with the release of the CCSS Follow-Up 5 (FU5) survey; therefore, FU5 is utilized as a recruitment screening tool. Individuals who fulfill the criteria listed above are sent the screening questions: (1) Have you been diagnosed with a skin cancer; (2) Have you seen a regular health care provider in the past 2 years, or do you plan to see one in the next year; (3) Do you have a mobile phone that can receive text messages; and, (4) Do you have access to a smartphone or tablet? Participants are eligible to participate in the ASK study if they answer ‘no’ to the first question and ‘yes’ to the remaining questions.

### Recruitment

The study protocol was reviewed and approved by the Institutional Review Board of Harvard TH Chan School of Public Health (approval number: IRB12-0002). Participants who meet study eligibility criteria will be invited to enroll. The invitation packet includes the invitation letter, Health Insurance Portability and Accountability Act (HIPAA) authorization, consent form, and the baseline survey.

Upon receipt of the completed survey and HIPAA authorization, the participant is officially enrolled in the study and randomized to one of three study arms. Within 7 days of randomization, each participant receives an enrollment packet that includes: a letter welcoming them to the study; print materials about their skin cancer risk, how to conduct skin self-examinations, an appointment checklist providing tips and suggestions about physician skin examinations for participants to reference when they go to see their doctor; and details about how to access the study website. Participants in Arm 3 also receive the dermatoscope and instructions for attaching it to their smartphone, using it, and uploading photos to the photo repository.

### Intervention components

The key intervention components are summarized in Table 1. The content of developed materials was partially influenced by Key Informant Interviews (n = 6, 50% female) conducted with members of the study cohort from which participants are being recruited. Common themes from these interviews regarding skin cancer knowledge and screening practices, attitudes about survivor status, preferences for receiving health information, and Internet and mobile phone connectivity and use were foundational in designing the communication strategy that guided development of the study’s name, logo, website, and print materials. A key development in the functionality and user-friendliness of the intervention is the added development of a specially designed mobile interface of the study website for convenient and functional access on mobile devices such as mobile phones and tablets. Provider intervention materials were guided by discussions with both primary care physicians and physician specialists. The study name/logo, website, educational print materials, and image repository were developed by the Health Communication Core of Dana-Farber Cancer Institute (www.healthcommcore.org) in collaboration with study staff.

**Table 1:** **Core intervention components of the three arms**

	**Intervention strategy**	**Content**	**Mediating variables addressed**
PAE	• 14 text messages	• 1 text: welcoming participants to the study	• PAM components:
		• 12 texts: reminders to conduct thorough skin self-examinations (TSSE), prompts to see a health care provider (HCP) for suspect lesions, messages highlighting key areas on targeting mediators and suggesting relevant website content (for example, demo of TSSE, risk factors for skin cancer)	
			Playing an active role in early detection
		• 1 text: reminding participants about the 18-month survey	Self-efficacy re TSSE and asking HCP for skin exam
	• Study website	• Information about skin cancer risk among survivors, and the key role that skin self-examinations and early detection (ED) play	
			Knowledge/awareness
			Taking action
			**•** Risk perception: re cancer status
		• Information about how to conduct a self-examination and clinical images illustrating what to look for	
		• Information about how to ask a HCP for a skin exam:	**•** Barrier reduction
		Printable checklist	
		Guide to discussion	
		• Information about preventive behaviors	
		• Interactive skin exam quizzes	
		• Tips for making ED a priority	
		• Lay summaries of research on the efficacy of skin exams	
		• Videos and links to additional resources about skin cancer	
PAE + MD (Additional components/Mediators addressed)	• Physician activation packet	• Introduction letter describing intervention and encouraging MDs to do a full-body skin examination at the patient’s next visit	**•** Self-efficacy to perform a skin examination on the patient
		• Guide to conducting a full skin cancer examination	
		• Information about survivors’ increased risk of skin cancer	
		• Access to the provider section of the ASK website, which houses additional resources including clinical images illustrating what to look for	
	• Study website	• Information about skin cancer risk among survivors, and the key roles that skin exams and early detection (ED) play	**•** Barrier reduction related to HCP provision of skin examination (for example, how to do exam; how to identify suspicious lesions)
		• Video demonstration of how to conduct a thorough clinical skin examination	
		• Summaries of research on the efficacy of skin exams	
		• Videos and links to additional resources regarding skin cancer	
		• Access to all participant website materials	
PAE + MD + TD	• Telederm	• Participant given dermatoscope attachment	**•** Barrier reduction decrease wait times for dermatology review of lesions
		• Website provides information about typical and atypical lesions	
		• As part of regular TSSE, participant takes photographs of suspect lesions and sends to study dermatologist	
		• Teledermoscopy report sent within 1 week, includes instructions to health care provider about needed follow-up or treatment	

Abbreviations: PAE, Patient Activation and Education, PAE + MD, PAE plus physician activation; PAE + MD + TD, PAE plus physician activation, plus teledermoscopy, PAM, Patient Activation Measure.

#### Arm 1: Patient Activation and Education

All participants receive educational print materials; access via individual login to the study website, which provides additional information, videos, and images of abnormal lesions; text messages designed to prompt use of the website and the target behaviors; and study staff contact information. After receipt of the introductory materials packet, participants are encouraged to use the study website for education about typical and atypical lesions, and then to examine themselves for any lesions of concern. It is anticipated that employment of the PAM will guide participants toward increased awareness of their risk of skin cancer while positively encouraging them to engage in recommended screening behaviors. Participants receive 14 separate text messages (Table 2 for examples) throughout the 18-month period designed to encourage them to: (1) carefully examine their skin for cancer with the aid of the study’s tools, such as pictorial diagrams and photographs of abnormal lesions for reference; (2) discuss concerns with and request skin exams from their physicians utilizing a checklist that they can print to bring to their physician visit or access on the mobile study site during their appointment; and, (3) develop a collaborative care plan (between the physician and participant) including common responsibility for monitoring and quickly following up on new and changing moles and lesions.

**Table 2:** **Select text messages sent to participants over the 12-month study and follow-up period**

**Type of message**	**Message content**
Early detection Physician exam	ASK! Your past radiation treatment increases your skin cancer risk. Checking your skin monthly and asking for a Dr.’s exam are vital to catch & treat it early.
Early detectionPhysician exam	ASK! Your doctor is more likely to examine your skin if they know of your skin cancer risk. Here’s how to ask for an exam & an appt checklist: bit.ly/
Early detectionPhysician exam	ASK! If your doctor refers you to a dermatologist or other doctor for a mole or area of concern on your skin, follow-up right away! Here’s why: bit.ly/
Early detectionWebsite/Educational	The ABCDE rule will help you find unusual moles during your monthly skin self-check. More on the ABCDE rule & pictures plus watch a video: bit.ly/
Early detection Educational	ASK! Melanoma, the most serious form of skin cancer, “writes its message in the skin for all to see”. Check yourself regularly for anything unusual & follow up!
Early detectionWebsite	Thanks for being a part of ASK! Do a total skin check monthly–carefully looking at your skin from head to toe and front to back. Here’s how: bit.ly/
Early detectionWebsite/Educational	ASK! Skin cancer can grow from existing moles and damaged skin. Get to know your skin. Check for any changes. You’ll get better each time. Tips: bit.ly/

#### Arm 2: Patient Activation and Education plus physician activation

In addition to the PAE elements, participants’ physicians are mailed physician activation materials. The goal of the physician activation intervention is to increase provider knowledge about the importance of early detection for survivors, with a particular focus on this specific population, motivate these providers to educate and screen patients, and to provide them with the skills and confidence needed to perform clinical examinations. Provider intervention materials include: (1) an introduction letter describing the intervention and examination recommendations; (2) information about increased risk of skin cancer for adult survivors treated with radiation and the role of primary care in early detection and treatment; (3) instructions on how to conduct a full skin cancer examination; and, (4) access to the study website, with specific information for health care professionals that contains resources and images to assist in conducting skin exams and identifying potential skin cancers. Providers are also given access to the new web-based curriculum guide developed by the INFORMED study of skin screening for primary care physicians [46,47]. Physicians are provided with a study 1-800 number should they have questions regarding the examination or follow-up care or need help ensuring an expedited referral to a dermatologist from the American Academy of Dermatology.

#### Arm 3: Patient Activation and Education plus physician activation and teledermoscopy

In addition to PAE + MD, Arm 3 participants receive a dermatoscope that permits acquisition of dermoscopic images through the participant’s smartphone camera and instructional materials for use of this attachment including a customized instructional video. Participants can take high-resolution photographs of suspect moles or lesions and upload these photos via a secure web portal for review by the study dermatologist. Participants are instructed to ask significant others to take photographs of back-of-body lesions. A letter with the results of the assessment of submitted photographs to the study website will be sent to the participant’s identified physician encouraging the physicians to emphasize the importance of monthly self-examinations and encouraging referral to a dermatologist for a clinical examination, if necessary. Participants are informed that they must contact their physicians to receive dermoscopy reports. Participants have access to teledermoscopy for 12 months after enrollment.

**Figure 2:**
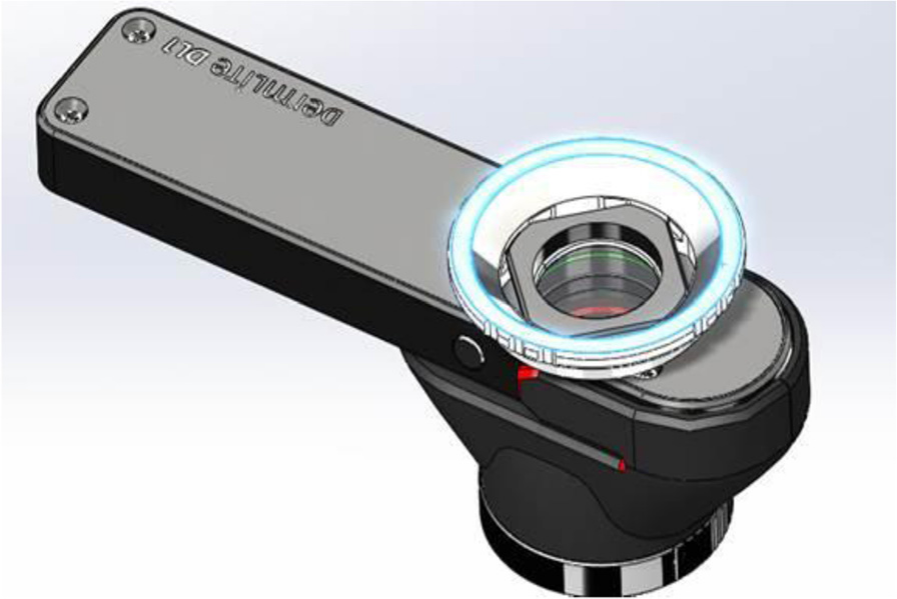
**DermLite lens attachment by 3Gen.**

The study staff, in cooperation with 3Gen, the manufacturer of the DermLite, developed written and video instructional materials to assist participants in effectively using the DermLite attachment and ensuring proper upload of photographs taken to be reviewed by the study dermatologist. The DermLite (Figure 2) is a portable dermatoscope that can be attached to smartphones and tablets and is primarily used for the examination of suspicious skin lesions. It consists of a magnification lens and light-emitting diode (LED) lighting that allows high-quality visualization of subsurface skin structures that are not typically visible to the naked eye. Advantages of this technology are that it can facilitate detection of skin cancers in the early stages of development and offers the possibility of increased convenience for both physicians and patients as it has the potential to be performed by the patient and sent remotely to a physician specialist. Print and online materials provided for Arm 3 participants include step-by-step instructions regarding the use of the DermLite, troubleshooting tips, and detailed instructions about uploading photos via the study website.

Another key element of the study is the development of the photo repository where participants in the PAE + MD + TD arm will upload DermLite photographs for review. Images can be uploaded from participants’ phones/tablets or the study website. When participants upload a photograph to the repository, they are asked a series of questions including: (1) date the photo was taken, (2) if they had previously submitted a photo of that particular mole, mark or spot, (3) location of the mole, mark, or spot (with a drop down menu of options to select from), and, (4) symptoms of the mole, mark or spot (with a drop down menu of options to select from). The photos will be reviewed by study staff to ensure acceptable quality and content before they are sent to the study dermatologist. The photographs will be reviewed by the study dermatologist and an individualized report and status will be generated for each photograph (for example, (a) morphology is suggestive of a benign lesion; (b) morphology is indeterminate and management will require evaluation and follow-up with a physician; (c) lesion has morphologic features suggestive of malignancy; and, (d) image is of insufficient quality - photograph needs to be retaken). The report will be sent to the corresponding participant’s physician. The participant will be instructed to contact his or her identified physician to receive the teledermoscopy report and to discuss follow-up measures.

### Tracking and follow-up

All participants will utilize a unique username and password to access the website. We are able to collect data on participants’ paths through the site, the amount of time spent on each page, total session time, and use of various components. Analytic software will be used to track aggregate website statistics. Participants will also be asked on the 12- and 18-month surveys to report their use of and experiences with the website. Study staff will utilize the photo repository database to track the number of photographs sent per participant as well as the time interval from the date the photograph was sent to the date that a report was processed. Additionally, on the 12- and 18-month surveys, participants will be asked if they brought the appointment checklist to their physician visits and the frequency with which they examined their skin, but found lesions of little concern, and chose not to use teledermoscopy (PAE + MD + TD arm only).

### Outcomes and measurements

#### Thorough skin self-examination (TSSE)

Self-report of TSSE has been validated [22,48], and as an outcome will be defined as performing at least one TSSE during the 2 months prior to the 12- and 18-month follow-up assessments. Participants will be asked how often in the prior 2 months they had performed a TSSE and if, in the 2 months prior to survey completion, they carefully examined each of 9 areas of the body (‘the front of your body from the waist up’, ‘the front of your thighs and legs’, ‘the bottom of your feet’, ‘your calves’, ‘the backs of your thighs’, ‘your buttocks’, ‘the lower parts of your back’, ‘your upper back’, and ‘your scalp’) [22,48]. Those who respond ‘yes’ to each of these nine questions will be considered to have performed TSSE.

#### Physician skin cancer examination

Completion of a physician skin exam will be assessed at baseline, 12 months, and 18 months by participant report and chart review. At each assessment time point, participants will be asked: ‘During the past 12 months, has your regular health care provider or your dermatologist carefully examined your whole body for any sign of skin cancer’, and whether the examination took place during a routinely scheduled visit or was prompted by a request from the participant. Participants’ self-report will be corroborated via chart review using a standard form submitted to the provider to assess each of the following: a skin examination has been performed, a skin self-exam has been recommended, and completion of a follow-up visit and the date for such a visit has been reported. Physician offices will be incentivized a total of $25.00 for review of the participant’s medical record related to the skin exam to be completed after the participant completes his/her 18-month survey.

#### Reduced time interval

We used the following assumptions from the literature: mean wait time (time between call for appointment and actual appointment) of 38.2 days (95% CI = (35.4, 41.0) [49]. Based on the proportion of individuals with atypical moles and changing moles, we assume that for each of the study groups, there will be an estimated 60 to 75 participants per 267 in each group who will seek care from their primary physician or dermatologist in the 18-month period for a suspect skin lesion. A clinically significant improvement in reduction of time between the first finding of a suspect lesion and diagnostic visit would be between 14 and 21 days.

#### Mediating variables

We will also examine the impact of the intervention on key mediating variables hypothesized to be related to behavior change, including: risk perception related to one’s prior cancer status and risk of skin cancer, self-efficacy to perform a TSSE and to ask a physician to perform a complete skin examination, barriers (such as not knowing what types of moles or lesions to look for), demographics, skin cancer risk factors, skin cancer knowledge, attitudes toward TSSE, and detection awareness of basic warning signs of melanoma and BCCs [48,50-52]. In addition, we will test for interactions between these variables and the PAM, looking at subscales for risk perception, self-efficacy, barriers, and awareness [53].

#### Economic impact measures

To estimate the economic impact of the intervention, at each assessment, we will examine participants’ visits with primary care providers and dermatologists, diagnostic procedures including biopsies and imaging, and treatment for newly-diagnosed skin conditions. This self-reported information will be verified and supplemented by data collected in chart reviews. We will use Medicare’s Direct Practice Expense and Resource Based Relative Value Scale (RBRVS) to estimate average unit costs for physician and laboratory services. A range of unit cost estimates will be evaluated in sensitivity analysis.

Our assessment of the downstream costs of the intervention, as well as the cost of the intervention itself, will allow performance of a limited cost-effectiveness analysis. We will estimate the cost per additional full-body skin cancer exam completed and the cost per additional skin cancer case detected, comparing the three intervention arms. The economic impact of the intervention will be evaluated using standard incremental cost-effectiveness analysis methods, and sensitivity analysis will be used to assess the impact of assumptions and uncertainty on results and conclusions [54,55].

### Power calculations

Sample size considerations are based on an equal allocation of participants to intervention arms. Our primary outcome focuses on increases in both physician screening and skin self-examination; the prevalence of screening and expected effect sizes are derived from rates of physician screening in the CCSS population [32,56]. Similar increases for self-screening were found in a trial focused on skin self-examination amongst the siblings of melanoma patients conducted by Geller and Emmons [50]. Assuming that baseline physician screening rates are 30% across all randomization groups, a 10 to 15% increase in physician screening rates would be considered a clinically significant improvement. Based on an estimated 25% attrition rate by month 18, we propose recruiting 801 subjects who will be evenly divided across the 3 intervention groups. Using a main effects model, the design has at least 80% power to detect a 15% difference across any of the arms even if one takes a conservative 0.025 threshold for statistical significance. This is based on a 2-degree of freedom chi-squared test with n = 200 in each arm, assuming the screening rate in the PAE arm is 30% and at least one of the other arms is 15% higher. The actual power in this scenario is 85%. We will use a conservative 0.025 threshold to account for multiple comparisons.

In the unexpected case that the PAE-alone arm is found to be superior to the other 2 arms, we will have a number of mechanisms in place to study this from our baseline, 12- and 18-month surveys. In particular, we will be asking many questions on physician screening of the participants in all three conditions. It could be that participants are deterred by added involvement of their physicians, although the literature shows that physician prompts are some of the greatest influences in increasing screening behaviors. We will also have a series of questions specific to Arm 3 participants to test if there has been any part of the addition of the technology component that was off-putting (for example, some participants who are either overwhelmed, do not understand how to utilize it, or perhaps do not trust the transmission of their personal health information).

## Discussion

Focus on the long-term health outcomes of survivors of childhood cancer is of vital public health significance, as their 5-year survival rates now exceed 80%. Survivors treated with radiation therapy are currently at increased risk of developing both melanoma and basal cell carcinoma; incidence of squamous cell carcinoma may also emerge as the population ages. In theory, early detection can save lives and reduce morbidity, in practice though, screening rates are sub-optimal [32,56]. Less than optimal screening rates may be driven by lack of awareness of risk and lack of knowledge of how to conduct skin examinations by patients and physicians alike. The ASK study will address many of these barriers by providing educational and motivational information for both survivors and physicians regarding skin examinations, prompts via text messaging for survivors, and print and online resources for patients and providers. Equally important, we will attempt to narrow the interval from first detection of suspected skin cancer to treatment through the added dimension of teledermoscopy, which provides highly accurate preliminary information about the morphology of a lesion and can, in turn, lead to expedited care if needed.

There are a number of potential limitations, including recall bias and social desirability bias, specific to one of the three study conditions. Although it is highly unlikely that PAE alone or PAE with added interventions would cause worse outcomes than standard-of-care, because there is not a control group with no PAE, the study will not be able to detect such potential outcomes. In addition, social desirability may incline some participants to answer questions in a way that they think will be considered favorable by others. For example, if participants see that study researchers promote monthly skin self-checks, some may over report this behavior to appear more compliant with the expectations and desires of the researchers. Potentially over reporting positive behavior and/or under reporting negative behavior could bias the data and lead to inaccurate conclusions. However, consideration was given to ensure that questions and response options were not phrased in a way that would incline participants to choose a response because they felt it was ‘correct’. Another possible limitation is that the study staff will not observe participant performance of skin self-examination; therefore, the accuracy of these exams will not be able to be validated. The same is true regarding the accuracy and thoroughness of physician skin exam reports although documentation of screening will be verified through chart review. However, we have provided numerous resources to instruct and guide both participants and physicians regarding how to perform thorough, accurate skin examinations in several mediums (for example, written instructions, step-by-step guides with illustrations, and video tutorials). An additional potential limitation is that physicians may perform clinical skin examinations but may not document them in their patients’ charts, resulting in underreporting of clinical skin exams.

There are many strengths to the current study, particularly regarding the use of innovative technology and methods. First, we are targeting the three primary factors needed to result in reduced risk of serious skin cancers: (1) patient skin self-examinations; (2) physician full-body skin exams; and (3) rapid access to dermatologist evaluation of worrisome lesions. Second, we are focusing on both patient activation, a very important but not previously studied component of increasing skin cancer early detection, as well as activating patients’ primary physicians to conduct skin exams while providing them with the educational information needed to do so effectively. Third, we are providing a subset of study participants with a dermatoscope that easily attaches to a variety of smartphones or tablets to provide high-quality photographs of suspect lesions that will maximize the quality of teledermoscopy and increase access to evaluation. Because this technology is rapidly evolving, it is likely that the cost of such devices will continue to decrease and become increasingly available to the general population. Finally, this study has been designed as a comparative effectiveness trial, which will isolate the effects of adding specific components to the patient activation intervention. The inclusion of an economic component is just one reason to call this study a comparative-effectiveness trial. We strongly considered employing a true control group that would receive no website education or text messages. Upon much reflection though, it was felt that the inclusion of such activities could be at once effective, easy to implement, and have a broad reach.

Having such information from patients in the PAE group aligns with basic principles of comparative effectiveness research that is ‘designed to inform health care decisions to prevent, diagnose and treat health conditions by providing evidence of the effectiveness, benefits, and harms of various treatment options generated from research studies comparing multiple ways to deliver health care’ [57,58], all key features of the current study. In addition, this study intends to translate and disseminate research findings to diverse stakeholders at its conclusion to inform patients, providers, and decision-makers about which interventions are most effective for patients under specific circumstances, another key feature of comparative effectiveness trials [57]. This study represents the first attempt that we are aware of to employ disseminable and scalable technologies to activate high-risk patients to examine their skin for cancer, seek physician exams, and obtain expedited treatment.

It is anticipated that results from this intervention will have important implications for childhood cancer survivors and other high-risk populations, including organ transplant recipients (>225,000 recipients) and first-degree relatives of melanoma patients (>2 million Americans), all of whom share strong deficits in skin self-examinations and receipt of physician examinations for skin cancer [50,59-62].

## Trial status

We have not completed patient recruitment at the time of submission.

## Abbreviations

ABCDE: Asymmetry, Border, Color, Diameter, Evolution
ASK: Advancing Survivors’ Knowledge
BCC: basal cell carcinoma
CCSS: Childhood Cancer Survivor Study
ED: Early Detection
HCP: Health Care Provider
HIPAA: Health Insurance Portability and Accountability Act
FU5: Childhood Cancer Survivor Study Follow-Up 5 survey
LED: light-emitting diode
PAE: Patient Activation and Education
PAE + MD: Patient Activation and Education plus physician activation
PAE + MD + TD: Patient Activation and Education plus physician activation, plus teledermoscopy
PAM: Patient Activation Measure
RBRVS: Resource Based Relative Value Scale
TSSE: thorough skin self-examination
US: United States
